# Ultrasmooth Organic Films Via Efficient Aggregation Suppression by a Low-Vacuum Physical Vapor Deposition

**DOI:** 10.3390/ma14237247

**Published:** 2021-11-27

**Authors:** Youngkwan Yoon, Jinho Lee, Seulgi Lee, Soyoung Kim, Hee Cheul Choi

**Affiliations:** Department of Chemistry, Pohang University of Science and Technology (POSTECH), Pohang 37673, Korea; yyk104@postech.ac.kr (Y.Y.); leejinho@postech.ac.kr (J.L.); letty523@postech.ac.kr (S.L.); ksy0457@postech.ac.kr (S.K.)

**Keywords:** organic films, vapor deposition, aggregation suppression, cooling rate

## Abstract

Organic thin films with smooth surfaces are mandated for high-performance organic electronic devices. Abrupt nucleation and aggregation during film formation are two main factors that forbid smooth surfaces. Here, we report a simple fast cooling (FC) adapted physical vapor deposition (FCPVD) method to produce ultrasmooth organic thin films through effectively suppressing the aggregation of adsorbed molecules. We have found that thermal energy control is essential for the spread of molecules on a substrate by diffusion and it prohibits the unwanted nucleation of adsorbed molecules. FCPVD is employed for cooling the horizontal tube-type organic vapor deposition setup to effectively remove thermal energy applied to adsorbed molecules on a substrate. The organic thin films prepared using the FCPVD method have remarkably ultrasmooth surfaces with less than 0.4 nm root mean square (RMS) roughness on various substrates, even in a low vacuum, which is highly comparable to the ones prepared using conventional high-vacuum deposition methods. Our results provide a deeper understanding of the role of thermal energy employed to substrates during organic film growth using the PVD process and pave the way for cost-effective and high-performance organic devices.

## 1. Introduction

Organic thin films are an effective material for device applications, such as organic light emitting diodes (OLEDs) and organic photovoltaics [1,2,3,4,5,6]. Developing methods for the deposition of organic thin film has been an important research topic in the material sciences fields for a long time [4,5,6,7,8]. One of many urgent issues regarding organic thin film is to achieve high quality—in terms of surface smoothness—that is largely hindered by the uncontrolled nucleation of target molecules or the formation of irregular aggregates [7,9,10,11,12]. Therefore, inhibiting abrupt nucleation and suppressing aggregates during film formation are two reasonable pathways for the formation of highly smooth films. For these, various approaches have been suggested by many research groups, including the Hu group, who demonstrates two-dimensional molecular crystals with smooth surfaces by space-confined self-assembly in solution [13,14], and the Chi group, who improves the surface roughness by using foreign particles in vapor phase [15].

The most conventional and standard method for the preparation of organic film is to deposit organic vapors under a high-vacuum environment (referred to as the ‘high-vacuum method’) [7,10,16,17,18,19]. A low-vacuum method, represented by physical vapor deposition (PVD) is an alternative [20,21,22,23]. Although the former is used as a standard technique for both industrial and academic research purposes, it is less advantageous than the latter mainly due to the cost required for a high-vacuum environment. Meanwhile, PVD suffers from the lack of understanding and controllability of nucleation and aggregation, which results in the loss of the cost-effectiveness advantage, as it runs under a low-vacuum environment [20,21,22]. 

Representing an important parameter, the effect of substrate temperature has been widely studied for high-vacuum methods. For example, Chiu and co-workers have reported the anomalous surface roughness change of tris-(8-hydroxyquinoline)aluminum at substrate temperatures between 90 and 120 °C [24]. They show that temperature change induces a thermal interconversion between the meridional and facial isomers, which eventually induces a higher degree of surface smoothness. Further, an amorphous to crystalline phase transformation of rubrene film has been reported by Im and co-workers [25]. Since the studies for the low-vacuum PVD method are relatively less popular, we focus on the parameters for reducing uncontrolled nucleation and aggregation during PVD.

In this report, we show that the aggregation of deposited molecules can be effectively suppressed by employing a fast cooling step in the PVD process (FCPVD). We demonstrate that the simple fast cooling step controls the thermal energy applied to a substrate to suppress nucleation and aggregation during the PVD process. Using this method, ultrasmooth thin films of four molecules—5,6,11,12-tetraphenyltetracene (rubrene), tris(8-hydroxyquinoline)aluminum (Alq_3_), N,N′-di(1-naphthyl)-N,N′-diphenyl-(1,1′-biphenyl)-4,4′-diamine (NPD), and [6,6]-phenyl-C_61_-butyric acid methyl ester (PCBM)—are obtained with less than 0.4 nm of root mean square (RMS) roughness. Further, it is confirmed that this method works for various substrates, including Si, Si/SiO_2_, quartz, mica, and indium tin oxide (ITO). The organic thin films formed through the FCPVD method in low-vacuum (10^−2^ Torr) conditions show lower RMS roughness than those films obtained using the high-vacuum method (10^−6^ Torr).

## 2. Materials and Methods

### 2.1. Growth of Organic Thin Films

**Figure 1:**
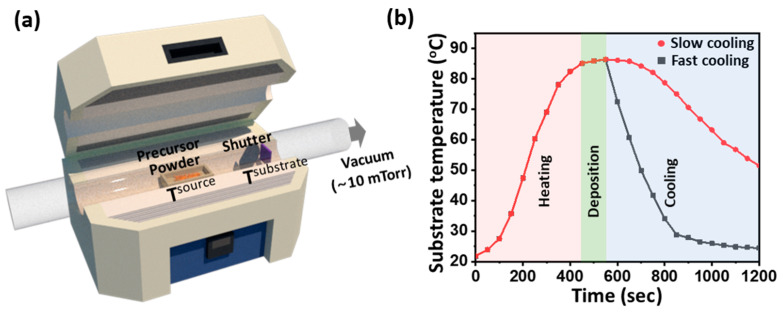
Experimental scheme and substrate temperature profile. (**a**) Schematic view of the horizontal PVD system used to obtain organic thin films. (**b**) Substrate temperature profiles of rubrene film using the SCPVD method (red) and the FCPVD method (grey) when the deposition time is 100 s.

All precursor powders were purchased from commercial sources (5,6,11,12-tetraphenylnaphthacene (rubrene, 98%, Sigma-Aldrich, Darmstadt, Germany), tris-(8-hydroxyquinoline)aluminum (Alq_3_, 98%, Sigma-Aldrich, Darmstadt, Germany), N,N′-di(1-naphthyl)-N,N′-diphenyl-(1,1′-biphenyl)-4,4′-diamine (NPD, 99%, Tokyo Chemical Industry, Kumagaya, Japan), and [6,6]-phenyl-C_61_-butyric acid methyl ester (PCBM, 99.5%, Tokyo Chemical Industry, Kumagaya, Japan) and used without further purification. FCPVD is performed using a horizontal tube furnace (TF55035C-1, Lindberg/Blue M, Waltham, Unites States) system (Figure 1a). Approximately 10 mg of precursor powder, loaded in a ceramic boat, is placed at the center of a tube furnace using a quartz protection tube. A substrate, where the organic vapors are to be deposited, is placed in the end region of the furnace where the temperature is naturally decreased. A PVD setup usually results in the end region of the furnace having a lower temperature than the center region. The furnace temperature is increased under low-vacuum conditions (~10^−2^ Torr) until the precursor powders are vaporized at the target source temperature (T^source^) and deposited on a substrate at the target substrate temperature (T^substrate^). The target temperature conditions are shown in Table S1. After reaching the target temperatures, the system is maintained for a designated deposition time (Table S1). The vapor supply is stopped after the designated deposition time using a shutter that is set between a precursor boat and a target substrate. The substrate temperature profiles of rubrene are shown in Figure 1b, and those of Alq_3_, NPD, and PCBM are shown in Figure S1 in the Supplementary Materials. The FCPVD method rapidly lowers substrate temperature by opening up the top cover of the furnace with the air blowing from an electric fan. Conversely, the slow cooling PVD (SCPVD) method lowers the substrate temperature gradually by simply turning off the furnace and closing the top cover. The deposition rate is approximately 1.2 nm/s, as measured from the film thickness. The film thickness is measured using AFM. The deposition time corresponds to the period during which the shutter is open.

The high-vacuum method is attempted using the same precursor powders through thermal evaporation under high-vacuum conditions (~10^−6^ Torr). Approximately 10 mg of precursor powder is used, with deposition rates of under 0.5 Å/s. Rubrene, Alq_3_, NPD, and PCBM are vaporized at 170 °C, 200 °C, 190 °C, and 280 °C, respectively, and deposited on Si/SiO_2_ substrates at T^substrate^ (Table S1) or room temperature (25 °C). 

### 2.2. Characterization of Organic Thin Films

The resulting films are analyzed using scanning electron microscopy (SEM, LYRA3, TESCAN, Brno, Czech) and atomic force microscopy (AFM, NanoScope IIIa, Santa Barbara, United States). Grazing incidence wide-angle X-ray scattering (GIWAXS, Pohang Accelerator Laboratory, Pohang, Korea) patterns of the resulting films are obtained from the 3C beamline at the Pohang Accelerator Laboratory (PAL) to examine the crystallinity of the films. All the measurements are made at room temperature.

### 2.3. Device Fabrication and Measurement

Organic films are deposited using PVD methods on 30 nm indium tin oxide (ITO)-coated glass substrates sonicated in a series of solvents and blown with N_2_ gas. The device structures are glass/ 30 nm ITO/ 120 nm Alq_3_/ 100 nm Al (electron-only device, EOD), glass/ 30 nm ITO/ 120 nm NPD/ 100 nm Al (hole-only device, HOD), and glass/ 30 nm ITO/ 120 nm NPD/ 120 nm Alq_3_/ 100 nm Al (heterojunction device). Al is deposited through e-beam evaporation (14-SN-004, SNTEK, Suwon, Korea) at 0.5 Å/s under ~10^−6^ Torr. Current density measurements are carried out at room temperature and in ambient conditions without any protective coatings. The results are obtained using a probe station (M6VC, MS TECH, Seoul, Korea) and a semiconductor analyzer (4200A-SCS, Keithley, Beaverton, United States).

## 3. Results and Discussion

### 3.1. Deposition of Ultrasmooth Organic Thin Films

**Figure 2:**
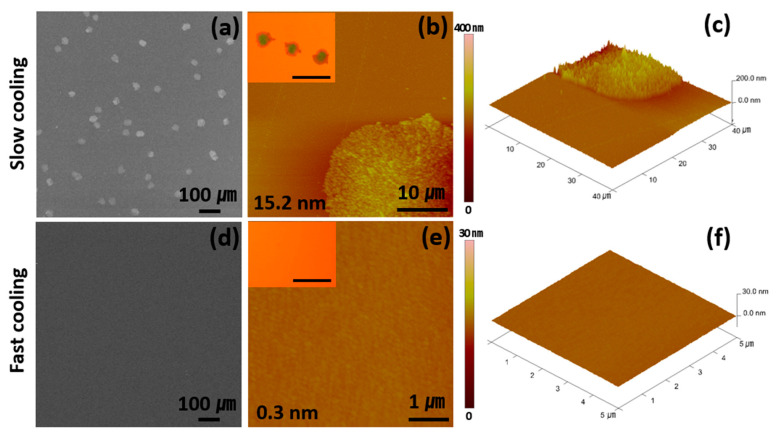
SEM and AFM images of rubrene films, depending on the cooling rate. The upper row images (**a**–**c**) and the lower row images (**d**–**f**) are obtained using SCPVD and FCPVD, respectively, when the deposition time is 100 s. The insets are OM images of each film (scale bar: 50 μm).

In this study, four organic molecules that are widely applied to organic devices—rubrene, Alq_3,_ NPD, and PCBM—are deposited using a horizontal PVD system (Figure 1a) [26,27,28,29,30,31,32,33]. The PVD system is designed to generate organic vapors at the center of the furnace at T^source^ and deposit them on a solid substrate located at the end of the furnace where the temperature is naturally lowered to T^substrate^. This system allows for the control of T^source^ and T^substrate^, correspondingly, which is important for the manipulation of the degrees of diffusion and the nucleation of adsorbed molecules on a substrate. We have first attempted the deposition of rubrene film using the PVD system. The rubrene films are prepared by following the conventional condition [34,35,36], i.e., rubrene powder is vaporized at a T^source^ of 250 °C and deposited on a Si/SiO_2_ substrate at a T^substrate^ of 85 °C, for various deposition times (10 s, 40 s, 70 s, 100 s, or 130 s). The thickness of each film is approximately 13 nm, 49 nm, 82 nm, 120 nm, and 155 nm, respectively. The thicknesses are measured using AFM. After deposition, the T^substrate^ is cooled down to room temperature by turning off the furnace and closing the top cover, which is referred to as the SCPVD method. The substrate temperature profile indicates that it takes about 600 s to lower the T^substrate^ from 85 to 50 °C (Figure 1b). The resulting films are very rough (over 10 nm of RMS roughness) with a high population of aggregated particles (Figure 2a–c, and S2) regardless of the deposition time, as frequently reported in many previous examples [34,35,37,38]. The presence of aggregated particles indicates that the aggregation of adsorbed molecules is dominant under this condition. 

There are two possible origins for the aggregation. The first is an insufficient diffusion of adsorbed molecules to evenly spread the molecules at T^substrate^ before they become aggregated, and the second is residual heat on substrates inducing nucleation, thus growing into aggregated particles [24,36,39,40,41,42]. To evaluate the latter possibility and diminish it, a fast cooling step that drops T^substrate^ down to room temperature in 300 s (Figure 1b) is employed by blowing air through the substrate region using an electric fan after the designated time of deposition. In high contrast to the result of the SCPVD method, smooth surfaces with remarkably reduced aggregates are obtained at a deposition time of 100 s (Figure 2d). To our great surprise, a remarkably ultrasmooth film of rubrene with 0.3 nm of RMS roughness is obtained (Figure 2e,f). This is a remarkable quality of surface roughness considering that the roughness of a bare Si/SiO_2_ substrate is 0.2 nm of RMS roughness (Figure S3). These results are notable, considering that the previously reported rubrene thin films have a roughness of approximately 0.9 nm [25,37,43]. Therefore, compared with previous studies, it is remarkable that rubrene thin films with 0.3 nm of RMS roughness can be formed through FCPVD in low-vacuum conditions. Furthermore, the thermal stability of rubrene films is tested using annealing rubrene films prepared through FCPVD at 70 °C under Ar for 3 h. The results show that the surface retains high smoothness at this condition (Figure S4), which is in high contrast to the previous result by Park et al. showing a rough surface upon similar treatment [25]. 

The successful formation of ultrasmooth rubrene film owes mainly to the adequate control of substrate temperature, allowing adsorbed molecules to diffuse on a substrate but prohibiting aggregation or nucleation [24,36,39,40,41,42]. When T^substrate^ is higher than 85 °C for more than 100 s during the slow cooling of the substrate (Figure 1b), very rough surfaces containing aggregates are formed, thus implying that nucleation starts at this condition (Figure 2a–c). This result shows that the high substrate temperature provides energy to overcome the activation energy for nucleation, causing aggregates. These aggregates are significantly reduced by lowering T^substrate^ down to room temperature through fast cooling (Figure 2d–f). However, low T^substrate^ is not the only parameter to consider in avoiding aggregates, as rough surfaces are still formed even at low T^substrate^ (40 °C) values (Figure S5); however, this occurs when the deposition time is not long enough (<100 s) (Figure S6). These results imply that appropriate thermal energy and time are required to evenly spread the molecules on a substrate. 

### 3.2. Versatility of the FCPVD Method Applied to Various Organic Molecules and Substrates

**Figure 3:**
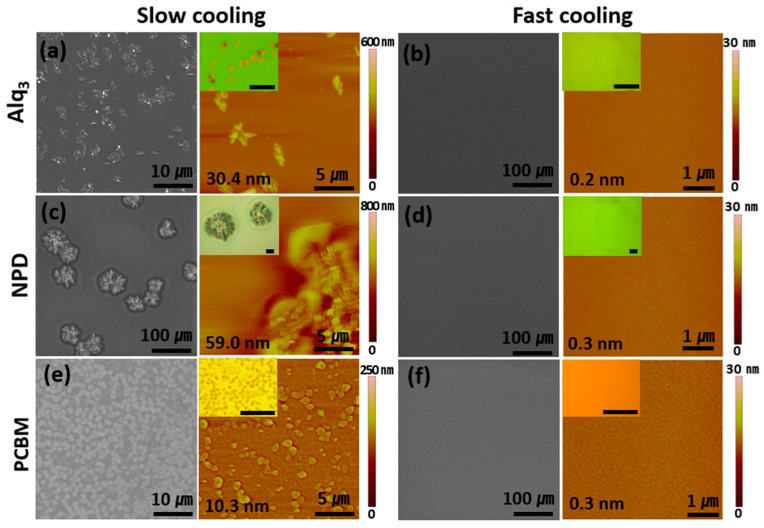
Various organic films, depending on the cooling rate of the substrate. SEM and AFM images of (**a**,**b**) Alq_3_ films, (**c**,**d**) NPD films, and (**e**,**f**) PCBM films, depending on the cooling rate. The films on the left side (**a**, **c,** and **e**) and those on the right side (**b**, **d,** and **f**) are obtained using SCPVD and FCPVD methods, respectively. The insets are optical microscope images of each film (scale bar: 10 μm).

Evaluating the versatility of the FCPVD method has been attempted with other organic molecules (Alq_3_, NPD, and PCBM), and all the tested molecules show similar results (Figure 3). While the films obtained through the SCPVD method have very rough surfaces (over 10 nm of RMS roughness) with a large population of aggregated particles, those films obtained through the FCPVD method have ultrasmooth surfaces (lower than 0.4 nm of RMS roughness) without particles or notable defects (Figure 3). Furthermore, the FCPVD method has been applied to various substrates, including Si, quartz, mica, and ITO substrates (Figure S3). These results show that the FCPVD method can be applied to various organic molecules and substrates by suppressing nucleation, resulting in ultrasmooth films. 

**Figure 4:**
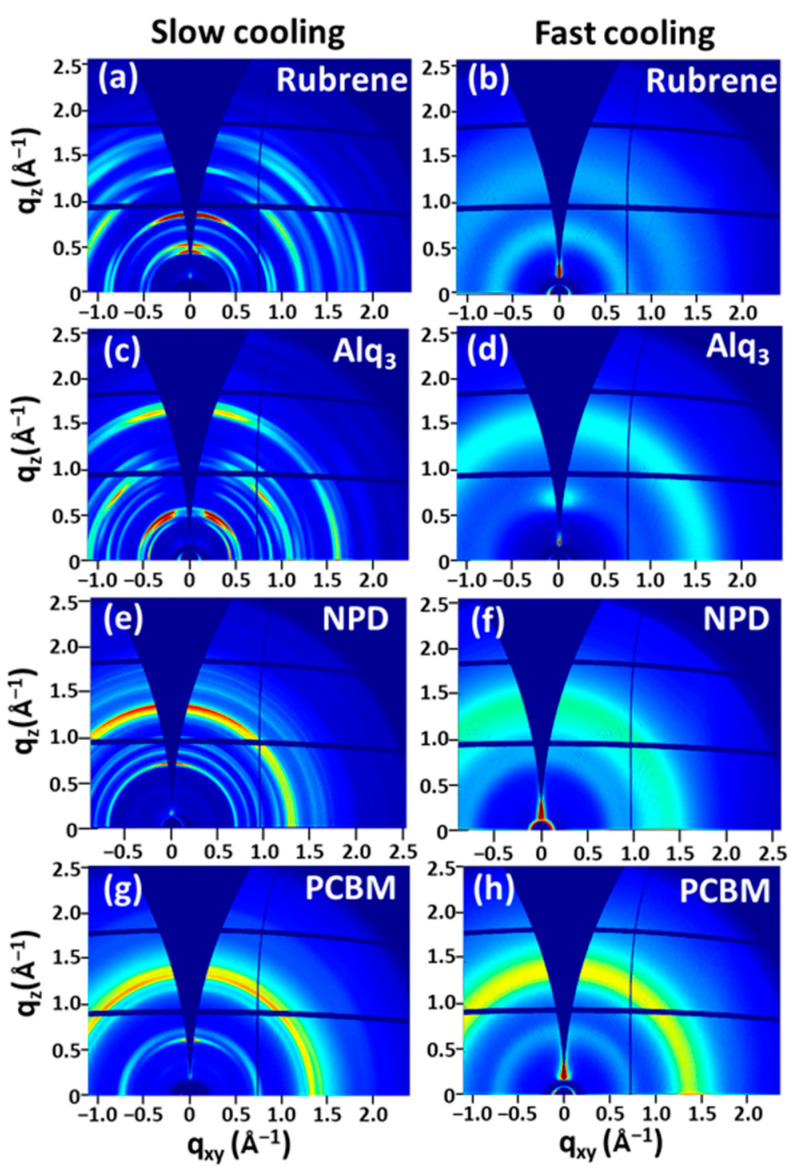
Structure analysis of organic thin films. GIWAXS patterns of (**a**,**b**) rubrene films, (**c**,**d**) Alq_3_ films, (**e**,**f**) NPD films, and (**g**,**h**) PCBM films grown using the SCPVD (left column) and FCPVD (right column) methods.

The successful nucleation suppression using the FCPVD method has been confirmed through grazing incidence wide-angle X-ray scattering (GIWAXS) analysis. These results indicate that the rough films are crystalline, while smooth films are amorphous (Figure 4). q_xy_ and q_z_ are the scattering vectors in the plane and out of the plane, respectively. The scattering intensities are represented by colors from high (red) to low (blue). The rubrene film obtained using the SCPVD method shows sharp and anisotropic scattering peaks; when assigned to a mixture of orthorhombic and triclinic phases, thermodynamically favorable crystalline forms from large aggregated particles (Figure 4a) [44,45]. Conversely, the rubrene film grown using the FCPVD method shows broad peaks at q ~0.68 Å^−1^ with isotropy, thus confirming the kinetically favorable amorphous phase (Figure 4b) [43]. Similar to rubrene films, Alq_3_, NPD, and PCBM films exhibit nucleation suppression using the FCPVD method (Figure 4d–h), while the films obtained using the SCPVD method exhibit sharp and anisotropic scattering peaks, indicating crystalline phases (Figure 4c–g). The deposited Alq_3_ and NPD films are characterized to be an α-phase and a triclinic phase, respectively, which are thermodynamically favorable forms [46,47]. Further, the PCBM film is well-matched with PCBM crystalline powder [48]. On the contrary, Alq_3_, NPD, and PCBM films grown using the FCPVD method show broad scattering peaks, confirming the kinetically favorable amorphous phase (Figure 4d–h) [17,49,50]. These results imply that the nucleation of adsorbed molecules is effectively suppressed by fast cooling the substrate and that this helps the formation of ultrasmooth organic thin films. 

### 3.3. Comparison of the FCPVD and High-Vacuum Method

**Figure 5:**
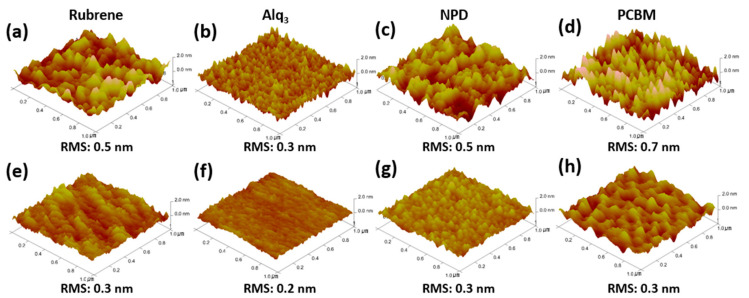
Surface morphologies of various organic thin films, depending on the deposition method. AFM images of (**a**,**e**) rubrene, (**b**,**f**) Alq_3_, (**c**,**g**) NPD, and (**d**,**h**) PCBM films on Si/SiO_2_ substrates. The films in the upper row of AFM images (**a**–**d**) and those in the lower row (**e**–**h**) are obtained using the high-vacuum method and FCPVD, respectively.

To evaluate the FCPVD method, rubrene, Alq_3_, NPD, and PCBM films prepared using FCPVD at 85 °C, 88 °C, 88 °C, and 122 °C of T^substrate^ (Table S1), respectively, are directly compared with the ones prepared using the high-vacuum method at the same T^substrate^ condition. The results show that all the films developed using FCPVD (Figure 5) are smoother than the ones developed using the high-vacuum method (Figure S7). Moreover, when depositing such films at room temperature using the high-vacuum method as a standard film growth condition [24,37,43,51,52], the results still show rougher surfaces than the thin films developed using FCPVD (Figure 5 and Table 1). This proves that the FCPVD method is a cost-effective and highly contending method for making ultrasmooth organic films.

### 3.4. Current Density-Voltage Characteristics of the Films

Among the target molecules in ultrasmooth films, Alq_3_ and NPD films are semiconductors in an amorphous phase and have been drawing great attention as electron transport layer and hole transport layer in OLED, respectively [53,54,55]. To further evaluate the FCPVD method, the quality of the film prepared using the FCPVD method has been examined with fabricated electron transport layer-only devices (EOD, ITO/Alq_3_/Al), hole transport layer-only devices (HOD, ITO/NPD/Al), and heterojunction devices (ITO/NPD/Alq_3_/Al)—which are the basic types of OLEDs—by analyzing the current density–voltage characteristics. All measurements are carried out at room temperature and in ambient conditions without any protective coatings. Before the measurements, the surface morphology of films is checked using AFM. Both Alq_3_ and NPD films on ITO substrates are very smooth (RMS: 0.2 nm and 0.4 nm, respectively) (Figure S3), and the roughness is maintained (RMS: 0.3 nm) in the heterojunction film, in which NPD and Alq_3_ thin films have been sequentially deposited on an ITO substrate (Figure S8). All devices fabricated using the FCPVD method exhibit typical current density–voltage curves without short circuits (Figure S9) [53,54,55]. Although the ultrasmooth films are obtained at low-vacuum conditions, the heterojunction device obtained using the FCPVD method shows a similar level of current density to the ones prepared in a high-vacuum system [53,54,55]. In contrast, all devices obtained using the SCPVD method are shorted because of the presence of aggregated particles in the film (Figure S10) [56,57]. These results support that the FCPVD method is effective for OLED fabrication, even with a cost-effective low-pressure system.

## 4. Conclusions

In summary, we show that the fast cooling of substrates during the PVD process results in the ultrasmooth surfaces of diverse organic films via aggregation suppression. The FCPVD method effectively removes residual heat on a substrate to prohibit nucleation, as it causes adsorbed molecules to diffuse to avoid nucleation. All the organic films grown using the FCPVD method have ultrasmooth surfaces, with less than 0.4 nm of RMS roughness on various substrates. Additionally, the organic thin films, obtained using the FCPVD method and made in low-vacuum conditions, exhibit smoother surfaces than ones formed using the conventional vapor deposition method in high-vacuum conditions. Considering the versatility of organic molecules and substrates and the usage of inexpensive low-vacuum conditions, this simple and effective method will contribute to the fabrication of low-cost, high-performance organic devices, as well as an understanding of organic thin film growth.

## Figures and Tables

**Table 1:** RMS roughness of various organic thin films, depending on the deposition method.

	Rubrene	Alq3	NPD	PCBM
High-vacuum method (10^−6^ Torr)	0.63 ± 0.075 nm	0.35 ± 0.040 nm	0.58 ± 0.055 nm	0.73 ± 0.038 nm
FCPVD (10−2 Torr)	0.33 ± 0.038 nm	0.22 ± 0.030 nm	0.35 ± 0.040 nm	0.35 ± 0.040 nm

RMS roughness of each film is obtained from 6 samples.

## Data Availability

The authors confirm that the data supporting the findings of this study are available within the article.

## Supplementary Materials

The following are available online at https://www.mdpi.com/article/10.3390/ma14237247/s1, Figure S1: substrate temperature profiles of Alq_3_, NPD, and PCBM depending on SCPVD method and FCPVD method, Figure S2: SEM and AFM images of rubrene films on Si/SiO_2_ substrate obtained by SCPVD method depending on various deposition times, Figure S3: AFM images of rubrene, Alq_3_, NPD, and PCBM films on Si/SiO_2_, Si, quartz, mica, and ITO substrates obtained by FCPVD method, Figure S4: AFM images of rubrene film by FCPVD, Figure S5: AFM images of rubrene films on the Si/SiO_2_ substrate obtained at low substrate temperature (40 °C) condition depending on deposition time, Figure S6: surface morphologies of rubrene films on the Si/SiO_2_ substrate obtained by FCPVD method depending on deposition time, Figure S7: optical microscope images of rubrene, Alq_3_, NPD, and PCBM films on Si/SiO_2_ substrates by the high-vacuum method, Figure S8: AFM images of heterojunction film in which NPD and Alq_3_ thin films were sequentially deposited on an ITO substrate by FCPVD, Figure S9: the current density-voltage characteristics of the devices prepared with ultrasmooth films obtained by FCPVD method, Figure S10: optical microscope images of NPD, Alq_3_, and NPD/Alq_3_ films on ITO substrates, Table S1: the target temperature conditions and deposition time for ultrasmooth films.
