# Supplementary material for: Ultrasmooth Organic Films Via Efficient Aggregation Suppression by a Low-Vacuum Physical Vapor Deposition
Source: Materials (Basel). 2021 Nov 27;14(23):7247. doi: 10.3390/ma14237247 (PMC8658267; doi:10.3390/ma14237247)
Supplement: Supplementary file 1 [file materials-14-07247-s001.zip › materials-1443836-supplementary.pdf]

Supplementary material

Ultrasmooth organic films via efficient aggregation suppression by a low-vacuum physical vapor deposition

Youngkwan Yoon, Jinho Lee, Seulgi Lee, Soyoung Kim, Hee Cheul Choi*

Department of Chemistry, Pohang University of Science and Technology (POSTECH), Pohang 37673, Republic of Korea

Corresponding authors. E-mail: choihc@postech.edu

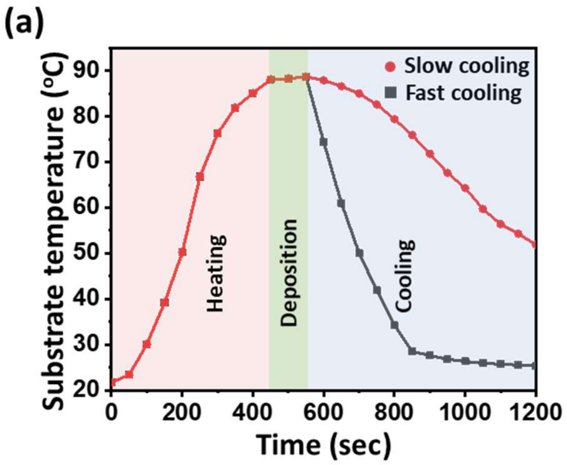

Figure S1. Substrate temperature profiles of (a) Alq_3, NPD, and (b) PCBM depending on SCPVD method (red) and FCPVD method (grey).

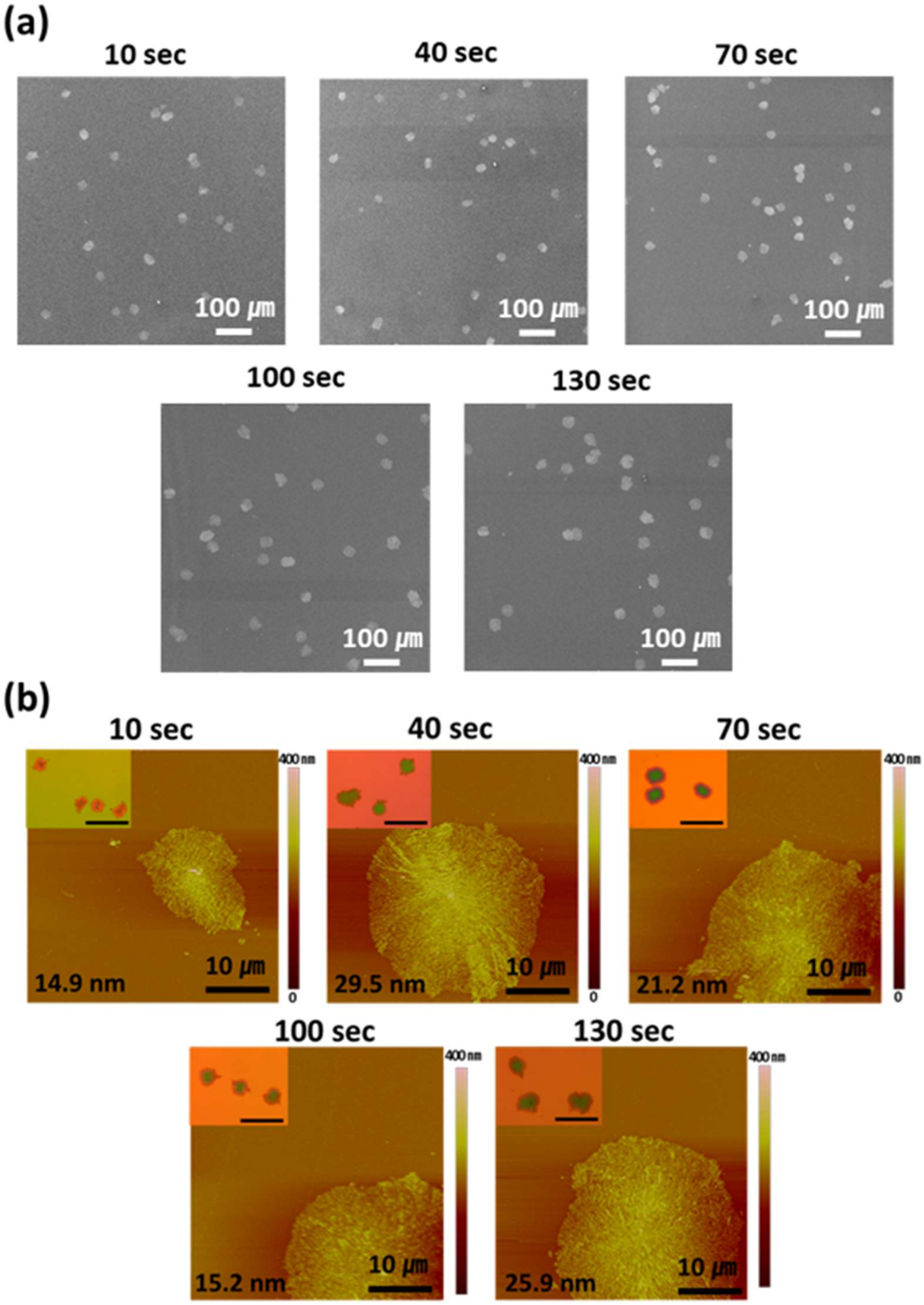

Figure S2. (a) SEM and (b) AFM images of rubrene films on Si/SiO_2 substrate obtained by SCPVD method depending on various deposition times.

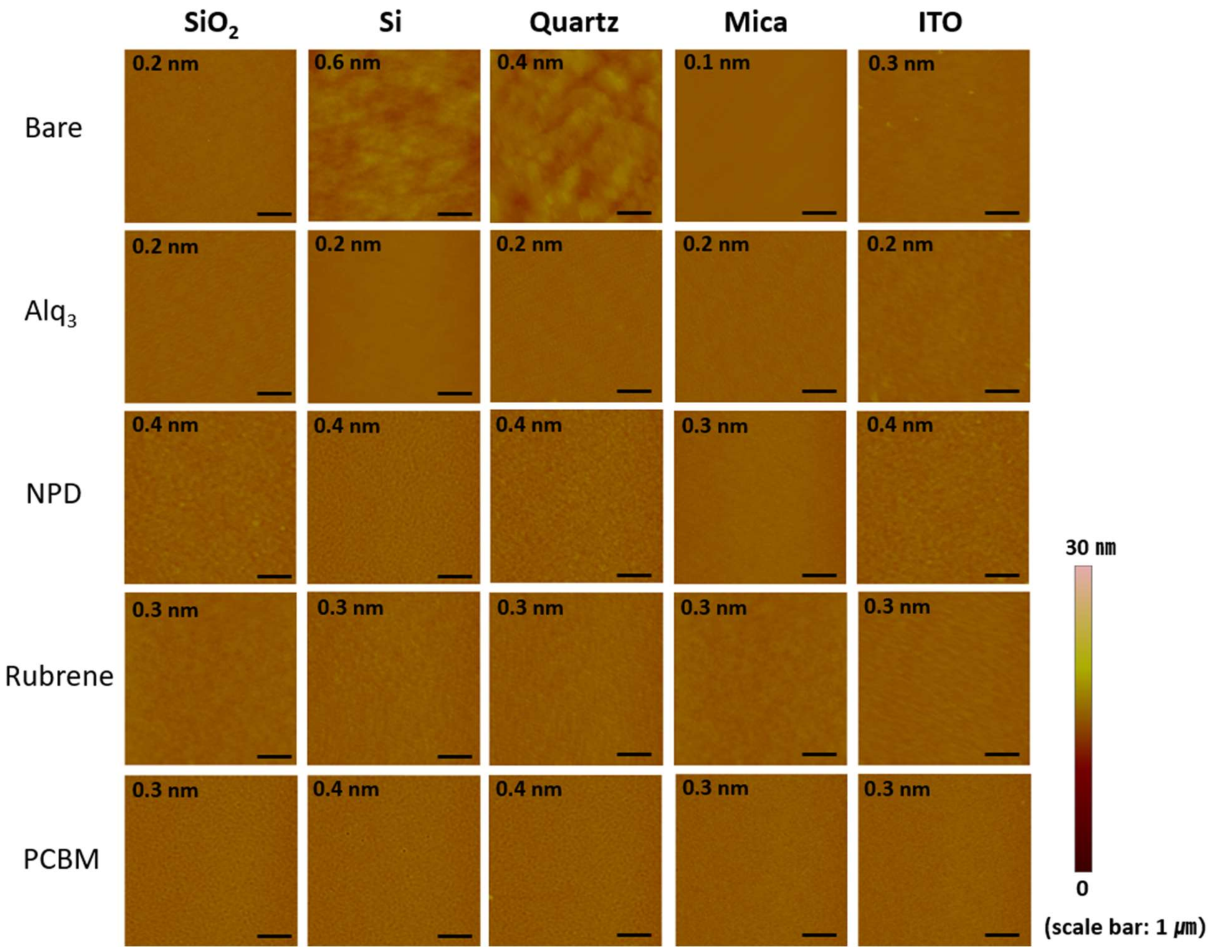

Figure S3. AFM images of rubrene, Alq_3, NPD, and PCBM films on Si/SiO_2, Si, quartz, mica, and ITO substrates obtained by FCPVD method.

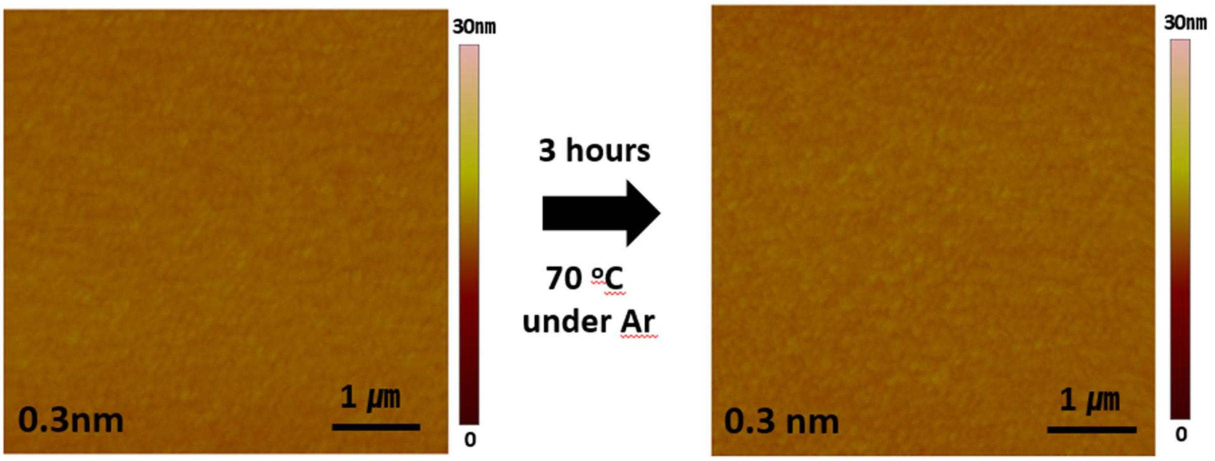

Figure S4. AFM images of rubrene film by FCPVD. The left image is as deposited rubrene film and the right image is the rubrene film annealed at 70 °C under Ar condition for 3 hours.

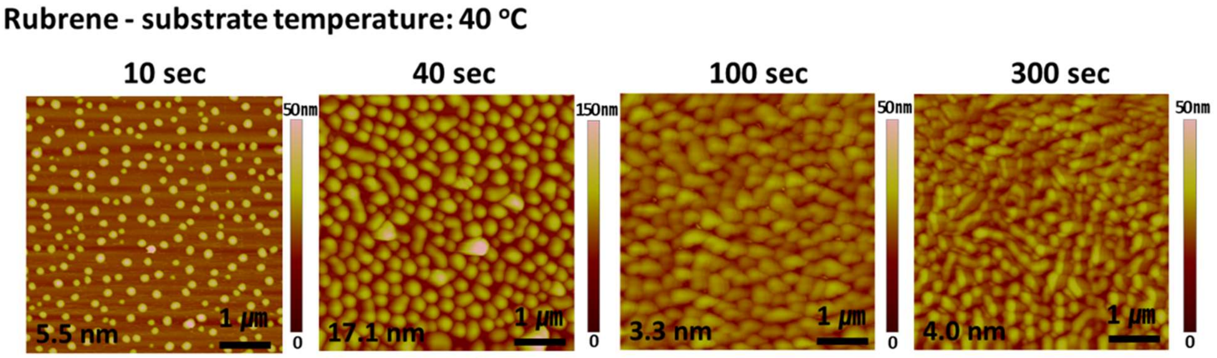

Figure S5. AFM images of rubrene films on the Si/SiO_2 substrate obtained at low substrate temperature (40 °C) condition depending on deposition time.

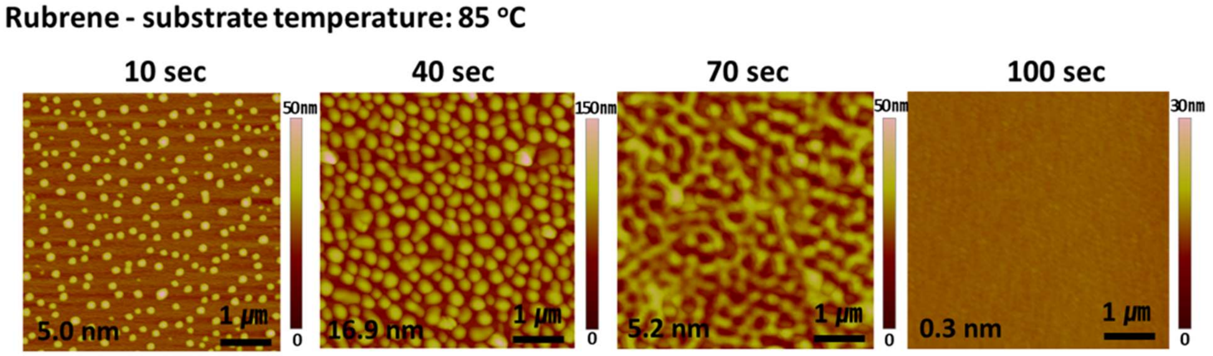

Figure S6. Surface morphologies of rubrene films on the Si/SiO_2 substrate obtained by FCPVD method depending on deposition time.

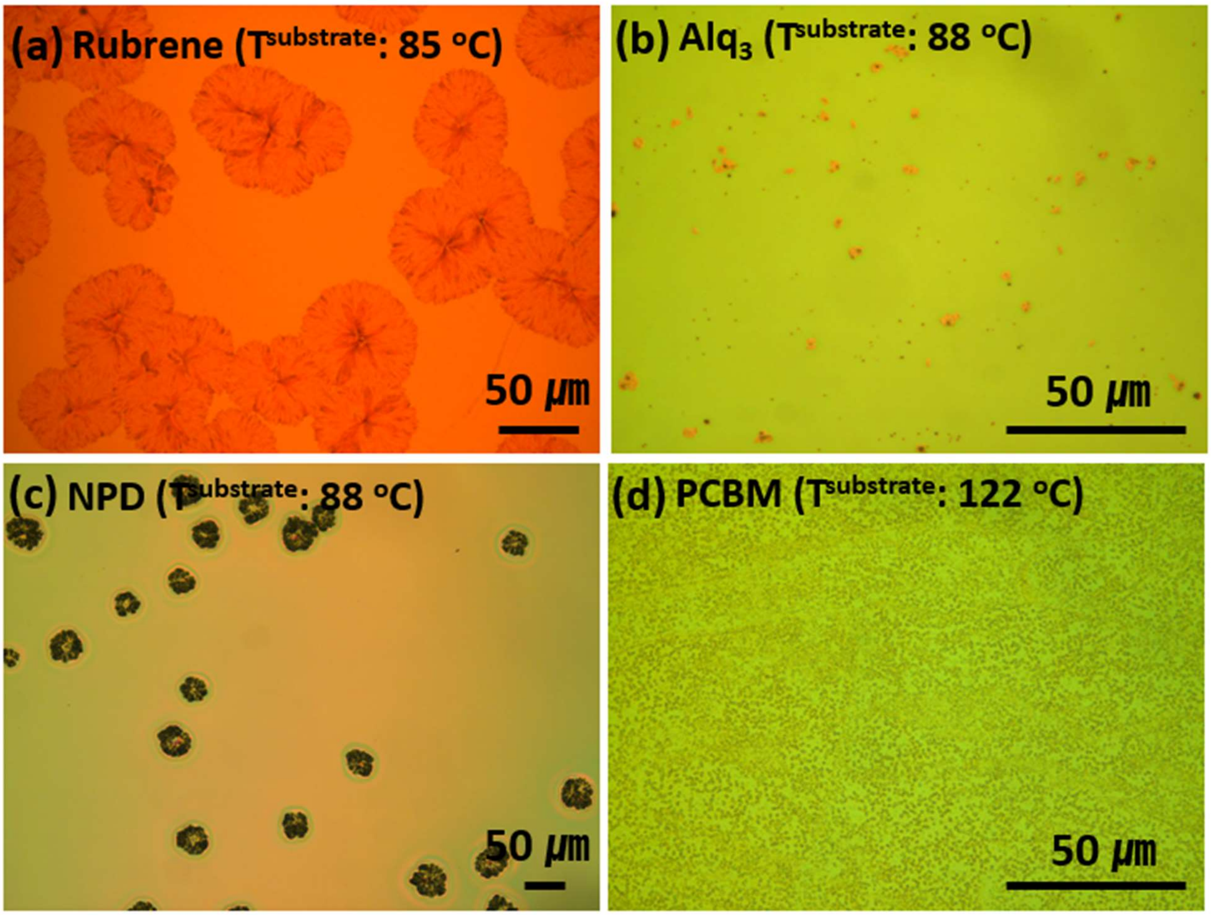

Figure S7. Optical microscope images of (a) rubrene, (b) Alq_3, (c) NPD, and (d) PCBM films on Si/SiO_2 substrates by the high-vacuum method.

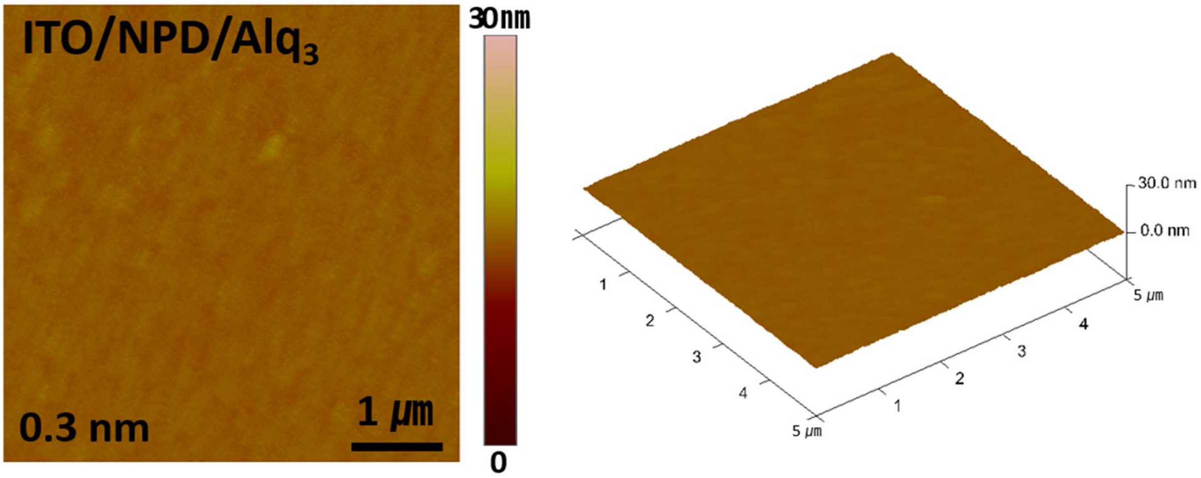

Figure S8. AFM images of heterojunction film in which NPD and Alq_3 thin films were sequentially deposited on an ITO substrate by FCPVD

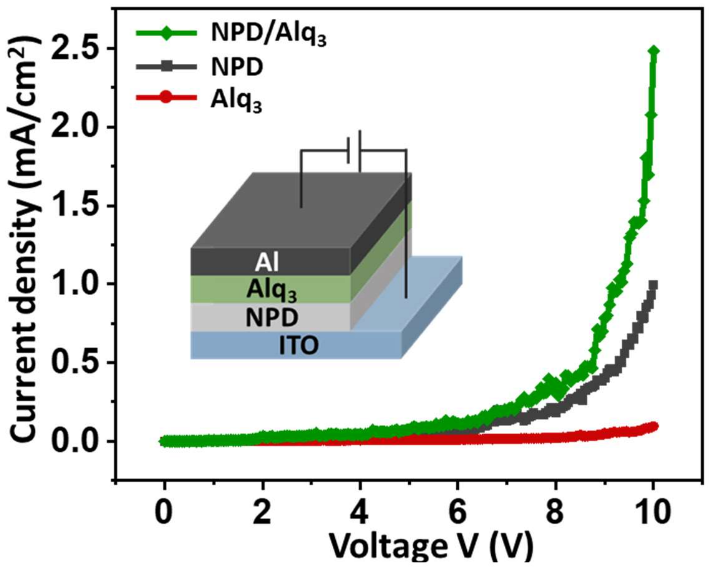

Figure S9. The current density-voltage characteristics of the devices prepared with ultrasmooth films obtained by FCPVD method. Heterojunction device (ITO/NPD/Alq_3/Al), HOD (ITO/NPD/Al), and EOD (ITO/Alq_3/Al) are represented as green, grey, and red lines respectively. The inset shows the device structure.

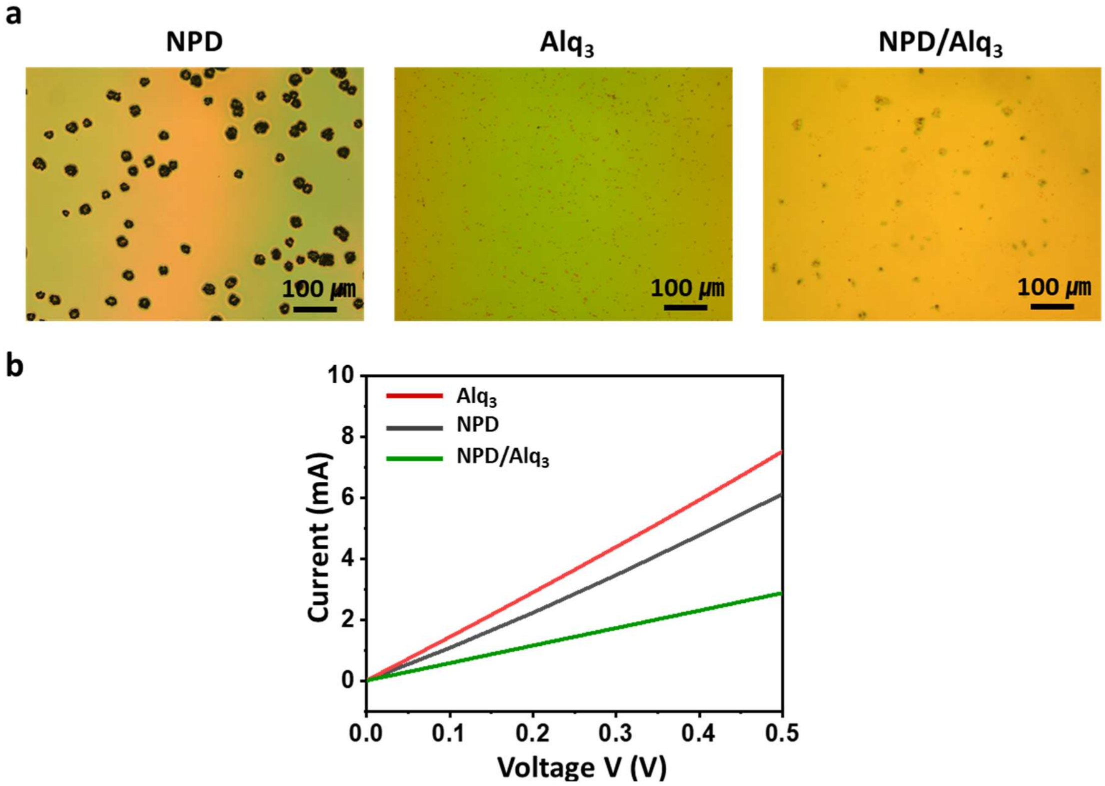

Figure S10. (**a**) Optical microscope images of NPD, Alq_3, and NPD/Alq_3 films on ITO substrates. (**b**) Current-voltage characteristics of the devices prepared with rough films obtained by SCPVD method. EOD (ITO/Alq_3/Al), HOD (ITO/NPD/Al), and heterojunction device (ITO/NPD/Alq_3/Al) are represented as red, grey, and green lines respectively.

Table S1. The target temperature conditions and deposition time for ultrasmooth films.

	T^{source}	$T^{substrate}$	Deposition time for ultrasmooth films
Rubrene	250 °C	85 °C	100 sec
Alq_3	260 °C	88 °C	110 sec
NPD	260 °C	88 °C	110 sec
PCBM	310 °C	122 °C	150 sec
